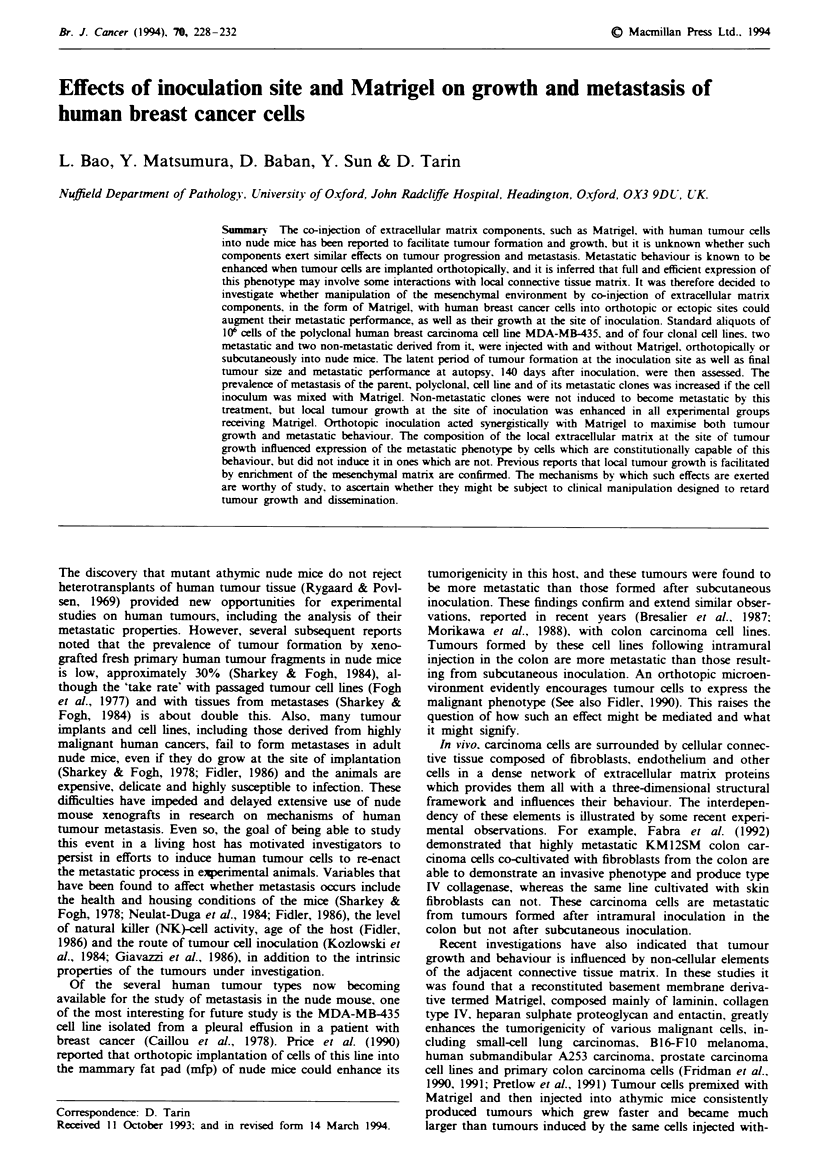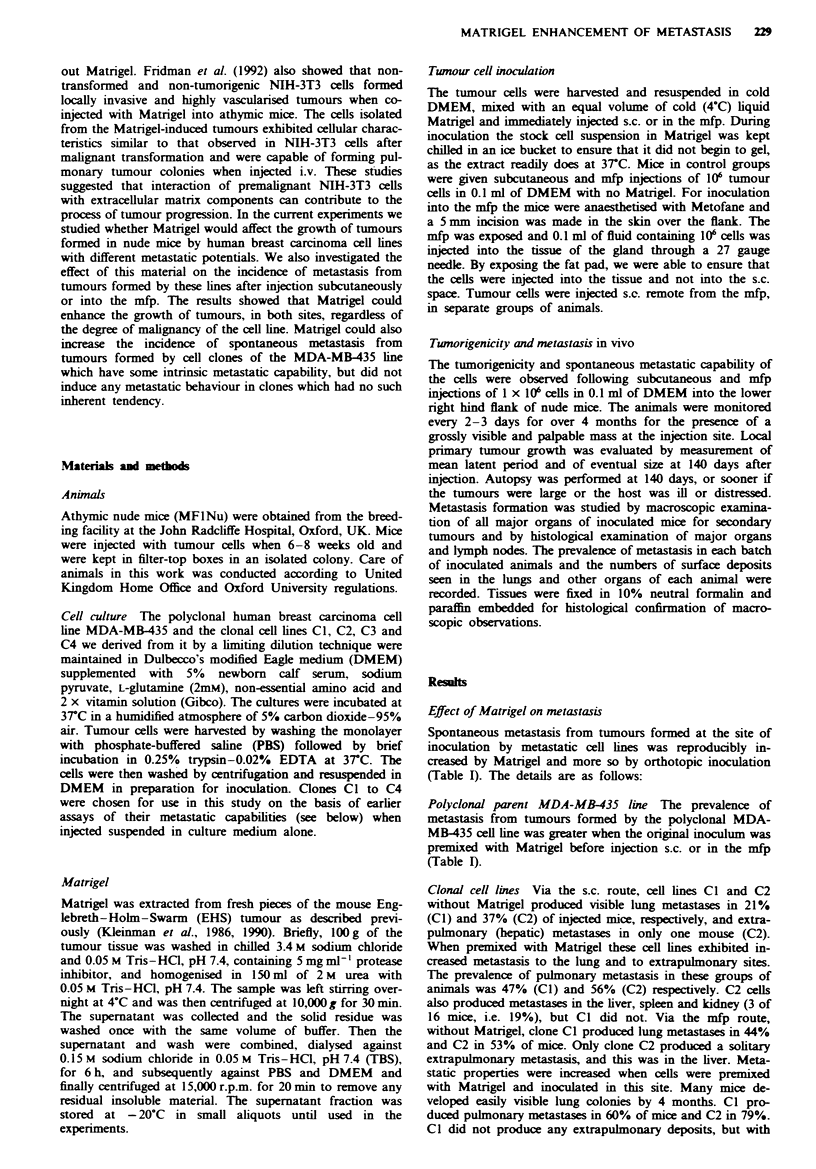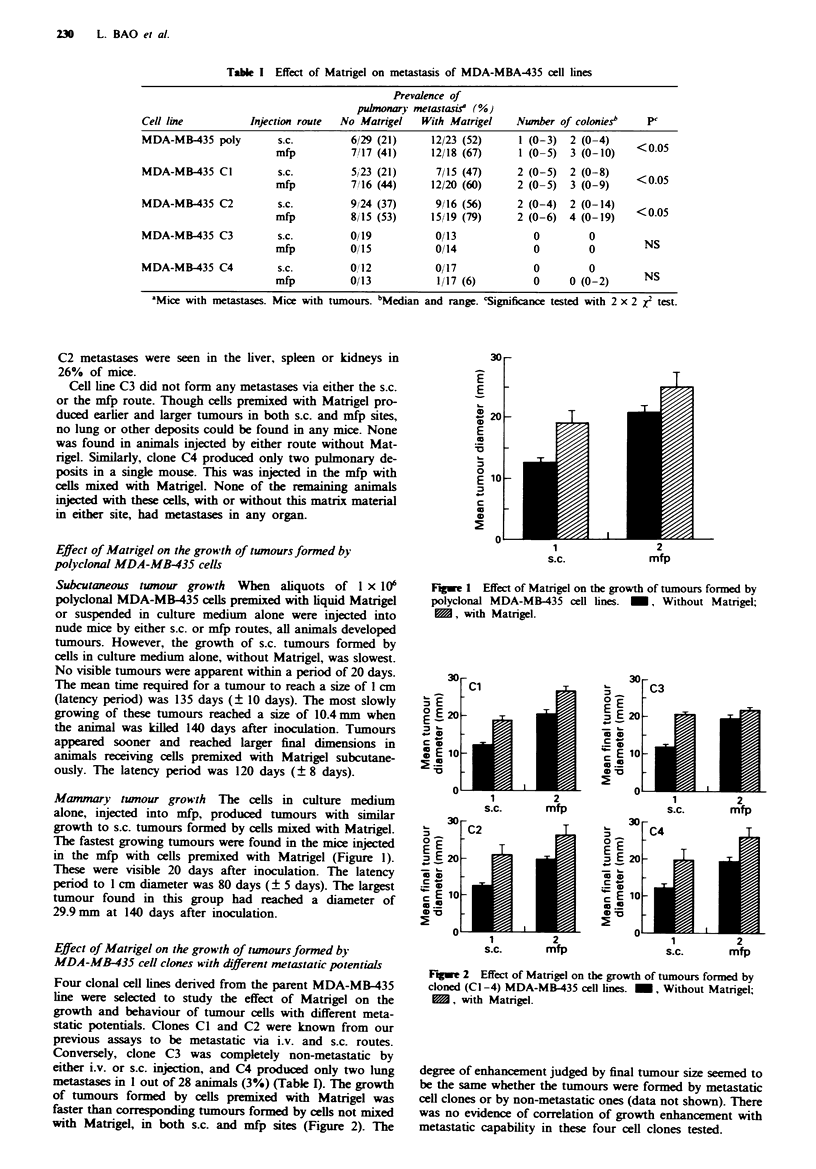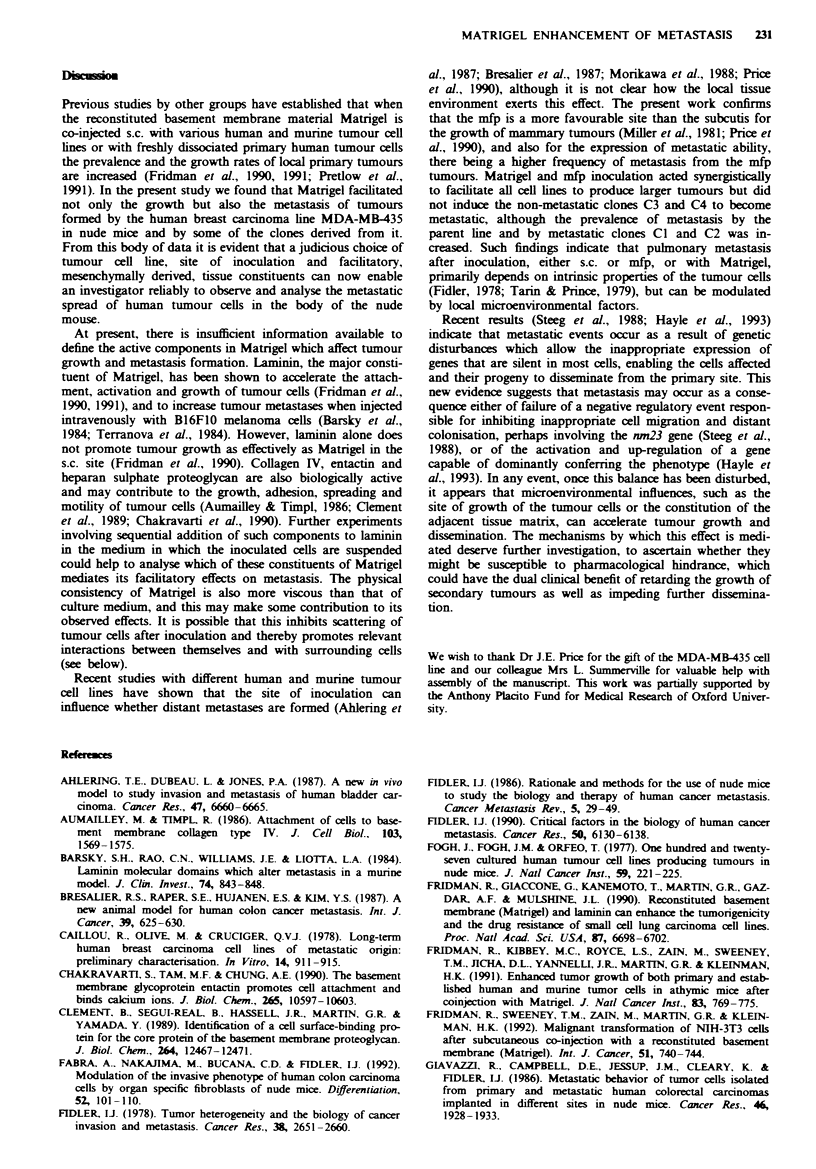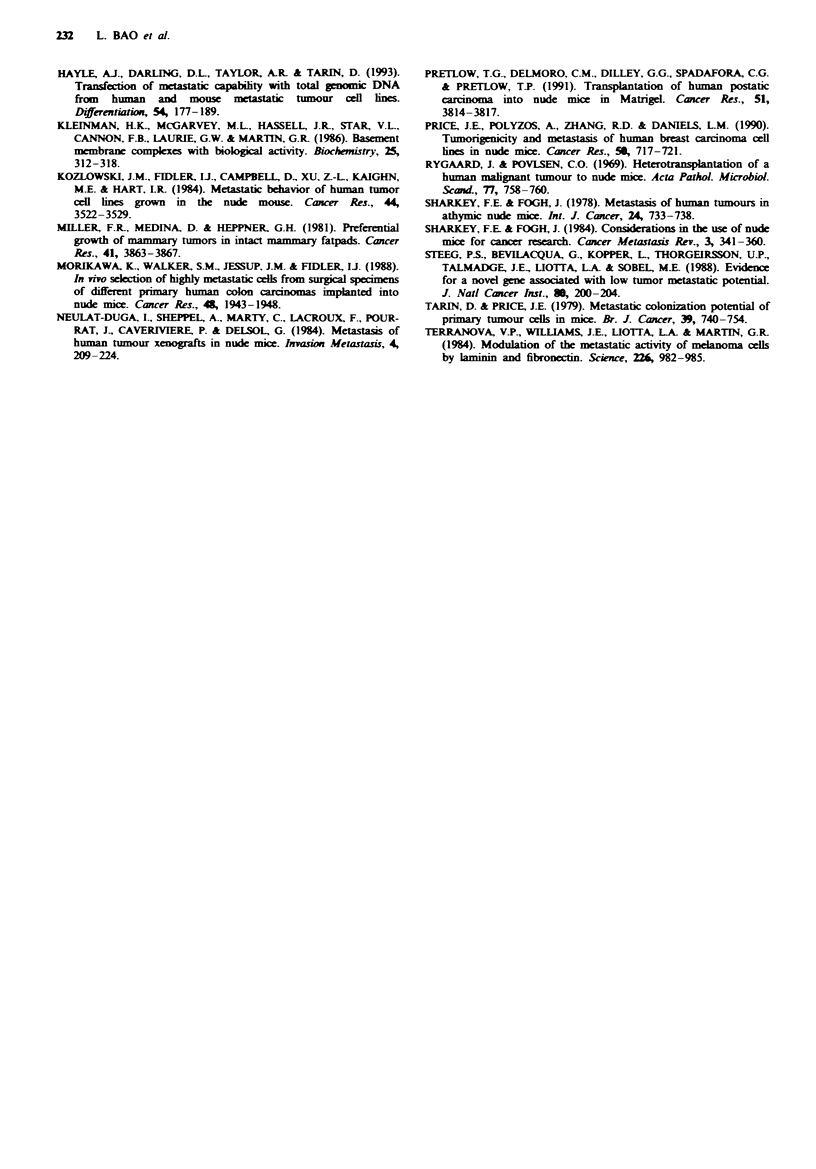# Effects of inoculation site and Matrigel on growth and metastasis of human breast cancer cells.

**DOI:** 10.1038/bjc.1994.284

**Published:** 1994-08

**Authors:** L. Bao, Y. Matsumura, D. Baban, Y. Sun, D. Tarin

**Affiliations:** Nuffield Department of Pathology, University of Oxford, John Radcliffe Hospital, Headington, UK.

## Abstract

The co-injection of extracellular matrix components, such as Matrigel, with human tumour cells into nude mice has been reported to facilitate tumour formation and growth, but it is unknown whether such components exert similar effects on tumour progression and metastasis. Metastatic behaviour is known to be enhanced when tumour cells are implanted orthotopically, and it is inferred that full and efficient expression of this phenotype may involve some interactions with local connective tissue matrix. It was therefore decided to investigate whether manipulation of the mesenchymal environment by co-injection of extracellular matrix components, in the form of Matrigel, with human breast cancer cells into orthotopic or ectopic sites could augment their metastatic performance, as well as their growth at the site of inoculation. Standard aliquots of 10(6) cells of the polyclonal human breast carcinoma cell line MDA-MB-435, and of four clonal cell lines, two metastatic and two non-metastatic derived from it, were injected with and without Matrigel, orthotopically or subcutaneously into nude mice. The latent period of tumour formation at the inoculation site as well as final tumour size and metastatic performance at autopsy, 140 days after inoculation, were then assessed. The prevalence of metastasis of the parent, polyclonal, cell line and of its metastatic clones was increased if the cell inoculum was mixed with Matrigel. Non-metastatic clones were not induced to become metastatic by this treatment, but local tumour growth at the site of inoculation was enhanced in all experimental groups receiving Matrigel. Orthotopic inoculation acted synergistically with Matrigel to maximise both tumour growth and metastatic behaviour. The composition of the local extracellular matrix at the site of tumour growth influenced expression of the metastatic phenotype by cells which are constitutionally capable of this behaviour, but did not induce it in ones which are not. Previous reports that local tumour growth is facilitated by enrichment of the mesenchymal matrix are confirmed. The mechanisms by which such effects are exerted are worthy of study, to ascertain whether they might be subject to clinical manipulation designed to retard tumour growth and dissemination.


Br. J. Cancer (1994), 70, 228-232 © Macmillan Press Ltd., 1994

### Effects of inoculation site and Matrigel on growth and metastasis of human breast cancer cells

L. Bao, Y. Matsumura, D. Baban, Y. Sun & D. Tarin

*Nuffield Department of Pathology, University of Oxford, John Radcliffe Hospital, Headington, Oxford, OX3 9DU, UK.*

**Summary** The co-injection of extracellular matrix components, such as Matrigel, with human tumour cells into nude mice has been reported to facilitate tumour formation and growth, but it is unknown whether such components exert similar effects on tumour progression and metastasis. Metastatic behaviour is known to be enhanced when tumour cells are implanted orthotopically, and it is inferred that full and efficient expression of this phenotype may involve some interactions with local connective tissue matrix. It was therefore decided to investigate whether manipulation of the mesenchymal environment by co-injection of extracellular matrix components, in the form of Matrigel, with human breast cancer cells into orthotopic or ectopic sites could augment their metastatic performance, as well as their growth at the site of inoculation. Standard aliquots of $10^6$ cells of the polyclonal human breast carcinoma cell line MDA-MB-435, and of four clonal cell lines, two metastatic and two non-metastatic derived from it, were injected with and without Matrigel, orthotopically or subcutaneously into nude mice. The latent period of tumour formation at the inoculation site as well as final tumour size and metastatic performance at autopsy, 140 days after inoculation, were then assessed. The prevalence of metastasis of the parent, polyclonal, cell line and of its metastatic clones was increased if the cell inoculum was mixed with Matrigel. Non-metastatic clones were not induced to become metastatic by this treatment, but local tumour growth at the site of inoculation was enhanced in all experimental groups receiving Matrigel. Orthotopic inoculation acted synergistically with Matrigel to maximise both tumour growth and metastatic behaviour. The composition of the local extracellular matrix at the site of tumour growth influenced expression of the metastatic phenotype by cells which are constitutionally capable of this behaviour, but did not induce it in ones which are not. Previous reports that local tumour growth is facilitated by enrichment of the mesenchymal matrix are confirmed. The mechanisms by which such effects are exerted are worthy of study, to ascertain whether they might be subject to clinical manipulation designed to retard tumour growth and dissemination.

The discovery that mutant athymic nude mice do not reject heterotransplants of human tumour tissue (Rygaard & Povlsen, 1969) provided new opportunities for experimental studies on human tumours, including the analysis of their metastatic properties. However, several subsequent reports noted that the prevalence of tumour formation by xenografted fresh primary human tumour fragments in nude mice is low, approximately 30% (Sharkey & Fogh, 1984), although the 'take rate' with passaged tumour cell lines (Fogh *et al.*, 1977) and with tissues from metastases (Sharkey & Fogh, 1984) is about double this. Also, many tumour implants and cell lines, including those derived from highly malignant human cancers, fail to form metastases in adult nude mice, even if they do grow at the site of implantation (Sharkey & Fogh, 1978; Fidler, 1986) and the animals are expensive, delicate and highly susceptible to infection. These difficulties have impeded and delayed extensive use of nude mouse xenografts in research on mechanisms of human tumour metastasis. Even so, the goal of being able to study this event in a living host has motivated investigators to persist in efforts to induce human tumour cells to re-enact the metastatic process in experimental animals. Variables that have been found to affect whether metastasis occurs include the health and housing conditions of the mice (Sharkey & Fogh, 1978; Neulat-Duga *et al.*, 1984; Fidler, 1986), the level of natural killer (NK)-cell activity, age of the host (Fidler, 1986) and the route of tumour cell inoculation (Kozlowski *et al.*, 1984; Giavazzi *et al.*, 1986), in addition to the intrinsic properties of the tumours under investigation.

Of the several human tumour types now becoming available for the study of metastasis in the nude mouse, one of the most interesting for future study is the MDA-MB-435 cell line isolated from a pleural effusion in a patient with breast cancer (Caillou *et al.*, 1978). Price *et al.* (1990) reported that orthotopic implantation of cells of this line into the mammary fat pad (mfp) of nude mice could enhance its tumorigenicity in this host, and these tumours were found to be more metastatic than those formed after subcutaneous inoculation. These findings confirm and extend similar observations, reported in recent years (Bresalier *et al.*, 1987; Morikawa *et al.*, 1988), with colon carcinoma cell lines. Tumours formed by these cell lines following intramural injection in the colon are more metastatic than those resulting from subcutaneous inoculation. An orthotopic microenvironment evidently encourages tumour cells to express the malignant phenotype (See also Fidler, 1990). This raises the question of how such an effect might be mediated and what it might signify.

*In vivo*, carcinoma cells are surrounded by cellular connective tissue composed of fibroblasts, endothelium and other cells in a dense network of extracellular matrix proteins which provides them all with a three-dimensional structural framework and influences their behaviour. The interdependency of these elements is illustrated by some recent experimental observations. For example, Fabra *et al.* (1992) demonstrated that highly metastatic KM12SM colon carcinoma cells co-cultivated with fibroblasts from the colon are able to demonstrate an invasive phenotype and produce type IV collagenase, whereas the same line cultivated with skin fibroblasts can not. These carcinoma cells are metastatic from tumours formed after intramural inoculation in the colon but not after subcutaneous inoculation.

Recent investigations have also indicated that tumour growth and behaviour is influenced by non-cellular elements of the adjacent connective tissue matrix. In these studies it was found that a reconstituted basement membrane derivative termed Matrigel, composed mainly of laminin, collagen type IV, heparan sulphate proteoglycan and entactin, greatly enhances the tumorigenicity of various malignant cells, including small-cell lung carcinomas, B16-F10 melanoma, human submandibular A253 carcinoma, prostate carcinoma cell lines and primary colon carcinoma cells (Fridman *et al.*, 1990, 1991; Pretlow *et al.*, 1991) Tumour cells premixed with Matrigel and then injected into athymic mice consistently produced tumours which grew faster and became much larger than tumours induced by the same cells injected with-

Correspondence: D. Tarin
Received 11 October 1993; and in revised form 14 March 1994.

out Matrigel. Fridman *et al.* (1992) also showed that non-transformed and non-tumorigenic NIH-3T3 cells formed locally invasive and highly vascularised tumours when co-injected with Matrigel into athymic mice. The cells isolated from the Matrigel-induced tumours exhibited cellular characteristics similar to that observed in NIH-3T3 cells after malignant transformation and were capable of forming pulmonary tumour colonies when injected i.v. These studies suggested that interaction of premalignant NIH-3T3 cells with extracellular matrix components can contribute to the process of tumour progression. In the current experiments we studied whether Matrigel would affect the growth of tumours formed in nude mice by human breast carcinoma cell lines with different metastatic potentials. We also investigated the effect of this material on the incidence of metastasis from tumours formed by these lines after injection subcutaneously or into the mfp. The results showed that Matrigel could enhance the growth of tumours, in both sites, regardless of the degree of malignancy of the cell line. Matrigel could also increase the incidence of spontaneous metastasis from tumours formed by cell clones of the MDA-MB-435 line which have some intrinsic metastatic capability, but did not induce any metastatic behaviour in clones which had no such inherent tendency.

### Materials and methods

### *Animals*

Athymic nude mice (MF1Nu) were obtained from the breeding facility at the John Radcliffe Hospital, Oxford, UK. Mice were injected with tumour cells when 6–8 weeks old and were kept in filter-top boxes in an isolated colony. Care of animals in this work was conducted according to United Kingdom Home Office and Oxford University regulations.

*Cell culture* The polyclonal human breast carcinoma cell line MDA-MB-435 and the clonal cell lines C1, C2, C3 and C4 we derived from it by a limiting dilution technique were maintained in Dulbecco's modified Eagle medium (DMEM) supplemented with 5% newborn calf serum, sodium pyruvate, L-glutamine (2mM), non-essential amino acid and 2 × vitamin solution (Gibco). The cultures were incubated at 37°C in a humidified atmosphere of 5% carbon dioxide–95% air. Tumour cells were harvested by washing the monolayer with phosphate-buffered saline (PBS) followed by brief incubation in 0.25% trypsin–0.02% EDTA at 37°C. The cells were then washed by centrifugation and resuspended in DMEM in preparation for inoculation. Clones C1 to C4 were chosen for use in this study on the basis of earlier assays of their metastatic capabilities (see below) when injected suspended in culture medium alone.

### *Matrigel*

Matrigel was extracted from fresh pieces of the mouse Engelbreth–Holm–Swarm (EHS) tumour as described previously (Kleinman *et al.*, 1986, 1990). Briefly, 100 g of the tumour tissue was washed in chilled 3.4 M sodium chloride and 0.05 M Tris–HCl, pH 7.4, containing 5 mg $ml^{-1}$ protease inhibitor, and homogenised in 150 ml of 2 M urea with 0.05 M Tris–HCl, pH 7.4. The sample was left stirring overnight at 4°C and was then centrifuged at 10,000 *g* for 30 min. The supernatant was collected and the solid residue was washed once with the same volume of buffer. Then the supernatant and wash were combined, dialysed against 0.15 M sodium chloride in 0.05 M Tris–HCl, pH 7.4 (TBS), for 6 h, and subsequently against PBS and DMEM and finally centrifuged at 15,000 r.p.m. for 20 min to remove any residual insoluble material. The supernatant fraction was stored at −20°C in small aliquots until used in the experiments.

### *Tumour cell inoculation*

The tumour cells were harvested and resuspended in cold DMEM, mixed with an equal volume of cold (4°C) liquid Matrigel and immediately injected s.c. or in the mfp. During inoculation the stock cell suspension in Matrigel was kept chilled in an ice bucket to ensure that it did not begin to gel, as the extract readily does at 37°C. Mice in control groups were given subcutaneous and mfp injections of $10^6$ tumour cells in 0.1 ml of DMEM with no Matrigel. For inoculation into the mfp the mice were anaesthetised with Metofane and a 5 mm incision was made in the skin over the flank. The mfp was exposed and 0.1 ml of fluid containing $10^6$ cells was injected into the tissue of the gland through a 27 gauge needle. By exposing the fat pad, we were able to ensure that the cells were injected into the tissue and not into the s.c. space. Tumour cells were injected s.c. remote from the mfp, in separate groups of animals.

### *Tumorigenicity and metastasis* in vivo

The tumorigenicity and spontaneous metastatic capability of the cells were observed following subcutaneous and mfp injections of $1 \times 10^6$ cells in 0.1 ml of DMEM into the lower right hind flank of nude mice. The animals were monitored every 2–3 days for over 4 months for the presence of a grossly visible and palpable mass at the injection site. Local primary tumour growth was evaluated by measurement of mean latent period and of eventual size at 140 days after injection. Autopsy was performed at 140 days, or sooner if the tumours were large or the host was ill or distressed. Metastasis formation was studied by macroscopic examination of all major organs of inoculated mice for secondary tumours and by histological examination of major organs and lymph nodes. The prevalence of metastasis in each batch of inoculated animals and the numbers of surface deposits seen in the lungs and other organs of each animal were recorded. Tissues were fixed in 10% neutral formalin and paraffin embedded for histological confirmation of macroscopic observations.

### Results

### *Effect of Matrigel on metastasis*

Spontaneous metastasis from tumours formed at the site of inoculation by metastatic cell lines was reproducibly increased by Matrigel and more so by orthotopic inoculation (Table I). The details are as follows:

*Polyclonal parent MDA-MB-435 line* The prevalence of metastasis from tumours formed by the polyclonal MDA-MB-435 cell line was greater when the original inoculum was premixed with Matrigel before injection s.c. or in the mfp (Table I).

*Clonal cell lines* Via the s.c. route, cell lines C1 and C2 without Matrigel produced visible lung metastases in 21% (C1) and 37% (C2) of injected mice, respectively, and extrapulmonary (hepatic) metastases in only one mouse (C2). When premixed with Matrigel these cell lines exhibited increased metastasis to the lung and to extrapulmonary sites. The prevalence of pulmonary metastasis in these groups of animals was 47% (C1) and 56% (C2) respectively. C2 cells also produced metastases in the liver, spleen and kidney (3 of 16 mice, i.e. 19%), but C1 did not. Via the mfp route, without Matrigel, clone C1 produced lung metastases in 44% and C2 in 53% of mice. Only clone C2 produced a solitary extrapulmonary metastasis, and this was in the liver. Metastatic properties were increased when cells were premixed with Matrigel and inoculated in this site. Many mice developed easily visible lung colonies by 4 months. C1 produced pulmonary metastases in 60% of mice and C2 in 79%. C1 did not produce any extrapulmonary deposits, but with

**Table I** Effect of Matrigel on metastasis of MDA-MBA-435 cell lines

| *Cell line* | *Injection route* | *Prevalence of pulmonary metastasis*[a] *(%)* *No Matrigel* | *With Matrigel* | *Number of colonies*[b] | | P[c] |
|---|---|---|---|---|---|---|
| MDA-MB-435 poly | s.c. | 6/29 (21) | 12/23 (52) | 1 (0-3) | 2 (0-4) | <0.05 |
| | mfp | 7/17 (41) | 12/18 (67) | 1 (0-5) | 3 (0-10) | |
| MDA-MB-435 C1 | s.c. | 5/23 (21) | 7/15 (47) | 2 (0-5) | 2 (0-8) | <0.05 |
| | mfp | 7/16 (44) | 12/20 (60) | 2 (0-5) | 3 (0-9) | |
| MDA-MB-435 C2 | s.c. | 9/24 (37) | 9/16 (56) | 2 (0-4) | 2 (0-14) | <0.05 |
| | mfp | 8/15 (53) | 15/19 (79) | 2 (0-6) | 4 (0-19) | |
| MDA-MB-435 C3 | s.c. | 0/19 | 0/13 | 0 | 0 | NS |
| | mfp | 0/15 | 0/14 | 0 | 0 | |
| MDA-MB-435 C4 | s.c. | 0/12 | 0/17 | 0 | 0 | NS |
| | mfp | 0/13 | 1/17 (6) | 0 | 0 (0-2) | |

[a]Mice with metastases. Mice with tumours. [b]Median and range. [c]Significance tested with 2 × 2 $\chi^2$ test.

C2 metastases were seen in the liver, spleen or kidneys in 26% of mice.

Cell line C3 did not form any metastases via either the s.c. or the mfp route. Though cells premixed with Matrigel produced earlier and larger tumours in both s.c. and mfp sites, no lung or other deposits could be found in any mice. None was found in animals injected by either route without Matrigel. Similarly, clone C4 produced only two pulmonary deposits in a single mouse. This was injected in the mfp with cells mixed with Matrigel. None of the remaining animals injected with these cells, with or without this matrix material in either site, had metastases in any organ.

### *Effect of Matrigel on the growth of tumours formed by polyclonal MDA-MB-435 cells*

*Subcutaneous tumour growth* When aliquots of $1 \times 10^6$ polyclonal MDA-MB-435 cells premixed with liquid Matrigel or suspended in culture medium alone were injected into nude mice by either s.c. or mfp routes, all animals developed tumours. However, the growth of s.c. tumours formed by cells in culture medium alone, without Matrigel, was slowest. No visible tumours were apparent within a period of 20 days. The mean time required for a tumour to reach a size of 1 cm (latency period) was 135 days (± 10 days). The most slowly growing of these tumours reached a size of 10.4 mm when the animal was killed 140 days after inoculation. Tumours appeared sooner and reached larger final dimensions in animals receiving cells premixed with Matrigel subcutaneously. The latency period was 120 days (± 8 days).

*Mammary tumour growth* The cells in culture medium alone, injected into mfp, produced tumours with similar growth to s.c. tumours formed by cells mixed with Matrigel. The fastest growing tumours were found in the mice injected in the mfp with cells premixed with Matrigel (Figure 1). These were visible 20 days after inoculation. The latency period to 1 cm diameter was 80 days (± 5 days). The largest tumour found in this group had reached a diameter of 29.9 mm at 140 days after inoculation.

### *Effect of Matrigel on the growth of tumours formed by MDA-MB-435 cell clones with different metastatic potentials*

Four clonal cell lines derived from the parent MDA-MB-435 line were selected to study the effect of Matrigel on the growth and behaviour of tumour cells with different metastatic potentials. Clones C1 and C2 were known from our previous assays to be metastatic via i.v. and s.c. routes. Conversely, clone C3 was completely non-metastatic by either i.v. or s.c. injection, and C4 produced only two lung metastases in 1 out of 28 animals (3%) (Table I). The growth of tumours formed by cells premixed with Matrigel was faster than corresponding tumours formed by cells not mixed with Matrigel, in both s.c. and mfp sites (Figure 2). The

**Figure 1** Effect of Matrigel on the growth of tumours formed by polyclonal MDA-MB-435 cell lines. ■, Without Matrigel; ▨, with Matrigel.

**Figure 2** Effect of Matrigel on the growth of tumours formed by cloned (C1-4) MDA-MB-435 cell lines. ■, Without Matrigel; ▨, with Matrigel.

degree of enhancement judged by final tumour size seemed to be the same whether the tumours were formed by metastatic cell clones or by non-metastatic ones (data not shown). There was no evidence of correlation of growth enhancement with metastatic capability in these four cell clones tested.

### Discussion

Previous studies by other groups have established that when the reconstituted basement membrane material Matrigel is co-injected s.c. with various human and murine tumour cell lines or with freshly dissociated primary human tumour cells the prevalence and the growth rates of local primary tumours are increased (Fridman *et al.*, 1990, 1991; Pretlow *et al.*, 1991). In the present study we found that Matrigel facilitated not only the growth but also the metastasis of tumours formed by the human breast carcinoma line MDA-MB-435 in nude mice and by some of the clones derived from it. From this body of data it is evident that a judicious choice of tumour cell line, site of inoculation and facilitatory, mesenchymally derived, tissue constituents can now enable an investigator reliably to observe and analyse the metastatic spread of human tumour cells in the body of the nude mouse.

At present, there is insufficient information available to define the active components in Matrigel which affect tumour growth and metastasis formation. Laminin, the major constituent of Matrigel, has been shown to accelerate the attachment, activation and growth of tumour cells (Fridman *et al.*, 1990, 1991), and to increase tumour metastases when injected intravenously with B16F10 melanoma cells (Barsky *et al.*, 1984; Terranova *et al.*, 1984). However, laminin alone does not promote tumour growth as effectively as Matrigel in the s.c. site (Fridman *et al.*, 1990). Collagen IV, entactin and heparan sulphate proteoglycan are also biologically active and may contribute to the growth, adhesion, spreading and motility of tumour cells (Aumailley & Timpl, 1986; Clement *et al.*, 1989; Chakravarti *et al.*, 1990). Further experiments involving sequential addition of such components to laminin in the medium in which the inoculated cells are suspended could help to analyse which of these constituents of Matrigel mediates its facilitatory effects on metastasis. The physical consistency of Matrigel is also more viscous than that of culture medium, and this may make some contribution to its observed effects. It is possible that this inhibits scattering of tumour cells after inoculation and thereby promotes relevant interactions between themselves and with surrounding cells (see below).

Recent studies with different human and murine tumour cell lines have shown that the site of inoculation can influence whether distant metastases are formed (Ahlering *et al.*, 1987; Bresalier *et al.*, 1987; Morikawa *et al.*, 1988; Price *et al.*, 1990), although it is not clear how the local tissue environment exerts this effect. The present work confirms that the mfp is a more favourable site than the subcutis for the growth of mammary tumours (Miller *et al.*, 1981; Price *et al.*, 1990), and also for the expression of metastatic ability, there being a higher frequency of metastasis from the mfp tumours. Matrigel and mfp inoculation acted synergistically to facilitate all cell lines to produce larger tumours but did not induce the non-metastatic clones C3 and C4 to become metastatic, although the prevalence of metastasis by the parent line and by metastatic clones C1 and C2 was increased. Such findings indicate that pulmonary metastasis after inoculation, either s.c. or mfp, or with Matrigel, primarily depends on intrinsic properties of the tumour cells (Fidler, 1978; Tarin & Prince, 1979), but can be modulated by local microenvironmental factors.

Recent results (Steeg *et al.*, 1988; Hayle *et al.*, 1993) indicate that metastatic events occur as a result of genetic disturbances which allow the inappropriate expression of genes that are silent in most cells, enabling the cells affected and their progeny to disseminate from the primary site. This new evidence suggests that metastasis may occur as a consequence either of failure of a negative regulatory event responsible for inhibiting inappropriate cell migration and distant colonisation, perhaps involving the *nm23* gene (Steeg *et al.*, 1988), or of the activation and up-regulation of a gene capable of dominantly conferring the phenotype (Hayle *et al.*, 1993). In any event, once this balance has been disturbed, it appears that microenvironmental influences, such as the site of growth of the tumour cells or the constitution of the adjacent tissue matrix, can accelerate tumour growth and dissemination. The mechanisms by which this effect is mediated deserve further investigation, to ascertain whether they might be susceptible to pharmacological hindrance, which could have the dual clinical benefit of retarding the growth of secondary tumours as well as impeding further dissemination.

We wish to thank Dr J.E. Price for the gift of the MDA-MB-435 cell line and our colleague Mrs L. Summerville for valuable help with assembly of the manuscript. This work was partially supported by the Anthony Placito Fund for Medical Research of Oxford University.

### References

AHLERING, T.E., DUBEAU, L. & JONES, P.A. (1987). A new *in vivo* model to study invasion and metastasis of human bladder carcinoma. *Cancer Res.*, **47**, 6660-6665.

AUMAILLEY, M. & TIMPL, R. (1986). Attachment of cells to basement membrane collagen type IV. *J. Cell Biol.*, **103**, 1569-1575.

BARSKY, S.H., RAO, C.N., WILLIAMS, J.E. & LIOTTA, L.A. (1984). Laminin molecular domains which alter metastasis in a murine model. *J. Clin. Invest.*, **74**, 843-848.

BRESALIER, R.S., RAPER, S.E., HUJANEN, E.S. & KIM, Y.S. (1987). A new animal model for human colon cancer metastasis. *Int. J. Cancer*, **39**, 625-630.

CAILLOU, R., OLIVE, M. & CRUCIGER, Q.V.J. (1978). Long-term human breast carcinoma cell lines of metastatic origin: preliminary characterisation. *In Vitro*, **14**, 911-915.

CHAKRAVARTI, S., TAM, M.F. & CHUNG, A.E. (1990). The basement membrane glycoprotein entactin promotes cell attachment and binds calcium ions. *J. Biol. Chem.*, **265**, 10597-10603.

CLEMENT, B., SEGUI-REAL, B., HASSELL, J.R., MARTIN, G.R. & YAMADA, Y. (1989). Identification of a cell surface-binding protein for the core protein of the basement membrane proteoglycan. *J. Biol. Chem.*, **264**, 12467-12471.

FABRA, A., NAKAJIMA, M., BUCANA, C.D. & FIDLER, I.J. (1992). Modulation of the invasive phenotype of human colon carcinoma cells by organ specific fibroblasts of nude mice. *Differentiation*, **52**, 101-110.

FIDLER, I.J. (1978). Tumor heterogeneity and the biology of cancer invasion and metastasis. *Cancer Res.*, **38**, 2651-2660.

FIDLER, I.J. (1986). Rationale and methods for the use of nude mice to study the biology and therapy of human cancer metastasis. *Cancer Metastasis Rev.*, **5**, 29-49.

FIDLER, I.J. (1990). Critical factors in the biology of human cancer metastasis. *Cancer Res.*, **50**, 6130-6138.

FOGH, J., FOGH, J.M. & ORFEO, T. (1977). One hundred and twenty-seven cultured human tumour cell lines producing tumours in nude mice. *J. Natl Cancer Inst.*, **59**, 221-225.

FRIDMAN, R., GIACCONE, G., KANEMOTO, T., MARTIN, G.R., GAZDAR, A.F. & MULSHINE, J.L. (1990). Reconstituted basement membrane (Matrigel) and laminin can enhance the tumorigenicity and the drug resistance of small cell lung carcinoma cell lines. *Proc. Natl Acad. Sci. USA*, **87**, 6698-6702.

FRIDMAN, R., KIBBEY, M.C., ROYCE, L.S., ZAIN, M., SWEENEY, T.M., JICHA, D.L., YANNELLI, J.R., MARTIN, G.R. & KLEINMAN, H.K. (1991). Enhanced tumor growth of both primary and established human and murine tumor cells in athymic mice after coinjection with Matrigel. *J. Natl Cancer Inst.*, **83**, 769-775.

FRIDMAN, R., SWEENEY, T.M., ZAIN, M., MARTIN, G.R. & KLEINMAN, H.K. (1992). Malignant transformation of NIH-3T3 cells after subcutaneous co-injection with a reconstituted basement membrane (Matrigel). *Int. J. Cancer*, **51**, 740-744.

GIAVAZZI, R., CAMPBELL, D.E., JESSUP, J.M., CLEARY, K. & FIDLER, I.J. (1986). Metastatic behavior of tumor cells isolated from primary and metastatic human colorectal carcinomas implanted in different sites in nude mice. *Cancer Res.*, **46**, 1928-1933.

HAYLE, A.J., DARLING, D.L., TAYLOR, A.R. & TARIN, D. (1993). Transfection of metastatic capability with total genomic DNA from human and mouse metastatic tumour cell lines. *Differentiation*, **54**, 177-189.

KLEINMAN, H.K., MCGARVEY, M.L., HASSELL, J.R., STAR, V.L., CANNON, F.B., LAURIE, G.W. & MARTIN, G.R. (1986). Basement membrane complexes with biological activity. *Biochemistry*, **25**, 312-318.

KOZLOWSKI, J.M., FIDLER, I.J., CAMPBELL, D., XU, Z.-L., KAIGHN, M.E. & HART, I.R. (1984). Metastatic behavior of human tumor cell lines grown in the nude mouse. *Cancer Res.*, **44**, 3522-3529.

MILLER, F.R., MEDINA, D. & HEPPNER, G.H. (1981). Preferential growth of mammary tumors in intact mammary fatpads. *Cancer Res.*, **41**, 3863-3867.

MORIKAWA, K., WALKER, S.M., JESSUP, J.M. & FIDLER, I.J. (1988). *In vivo* selection of highly metastatic cells from surgical specimens of different primary human colon carcinomas implanted into nude mice. *Cancer Res.*, **48**, 1943-1948.

NEULAT-DUGA, I., SHEPPEL, A., MARTY, C., LACROUX, F., POURRAT, J., CAVERIVIERE, P. & DELSOL, G. (1984). Metastasis of human tumour xenografts in nude mice. *Invasion Metastasis*, **4**, 209-224.

PRETLOW, T.G., DELMORO, C.M., DILLEY, G.G., SPADAFORA, C.G. & PRETLOW, T.P. (1991). Transplantation of human postatic carcinoma into nude mice in Matrigel. *Cancer Res.*, **51**, 3814-3817.

PRICE, J.E., POLYZOS, A., ZHANG, R.D. & DANIELS, L.M. (1990). Tumorigenicity and metastasis of human breast carcinoma cell lines in nude mice. *Cancer Res.*, **50**, 717-721.

RYGAARD, J. & POVLSEN, C.O. (1969). Heterotransplantation of a human malignant tumour to nude mice. *Acta Pathol. Microbiol. Scand.*, **77**, 758-760.

SHARKEY, F.E. & FOGH, J. (1978). Metastasis of human tumours in athymic nude mice. *Int. J. Cancer*, **24**, 733-738.

SHARKEY, F.E. & FOGH, J. (1984). Considerations in the use of nude mice for cancer research. *Cancer Metastasis Rev.*, **3**, 341-360.

STEEG, P.S., BEVILACQUA, G., KOPPER, L., THORGEIRSSON, U.P., TALMADGE, J.E., LIOTTA, L.A. & SOBEL, M.E. (1988). Evidence for a novel gene associated with low tumor metastatic potential. *J. Natl Cancer Inst.*, **80**, 200-204.

TARIN, D. & PRICE, J.E. (1979). Metastatic colonization potential of primary tumour cells in mice. *Br. J. Cancer*, **39**, 740-754.

TERRANOVA, V.P., WILLIAMS, J.E., LIOTTA, L.A. & MARTIN, G.R. (1984). Modulation of the metastatic activity of melanoma cells by laminin and fibronectin. *Science*, **226**, 982-985.